# Predicting Response to Anthracyclines in Ovarian Cancer

**DOI:** 10.3390/ijerph19074260

**Published:** 2022-04-02

**Authors:** Annamaria Ferrero, Martina Borghese, Stefano Restaino, Andrea Puppo, Giuseppe Vizzielli, Nicoletta Biglia

**Affiliations:** 1 Academic Department of Gynaecology and Obstetrics, Mauriziano Hospital, 10128 Torino, Italy; nicoletta.biglia@unito.it; 2 Department of Gynecology and Obstetrics, Santa Croce and Carle Hospital, 12100 Cuneo, Italy; martina.borghese1989@gmail.com (M.B.); puppo.a@ospedale.cuneo.it (A.P.); 3 Obstetrics and Gynecology Unit, Department of Obstetrics, Gynecology and Pediatrics, Department of Medical Area DAME, Udine University Hospital, 33100 Udine, Italy; restaino.stefano@gmail.com; 4 Clinic of Obstetrics and Gynaecology, Department of Medical Area (DAME), University of Udine, “Santa Maria della Misericordia” Hospital, Azienda Sanitaria Ospedaliera Friuli Centrale, 33100 Udine, Italy; giuseppe.vizzielli@uniud.it

**Keywords:** anthracyclines, pegylated liposomal doxorubicin, BRCA mutation, chemotherapy, immunohistochemistry, immunotherapy, organoids, ovarian cancer, p53, response rate

## Abstract

(1) Background: Anthracyclines are intriguing drugs, representing one of the cornerstones of both first and subsequent-lines of chemotherapy in ovarian cancer (OC). Their efficacy and mechanisms of action are related to the hot topics of OC clinical research, such as BRCA status and immunotherapy. Prediction of response to anthracyclines is challenging and no markers can predict certain therapeutic success. The current narrative review provides a summary of the clinical and biological mechanisms involved in the response to anthracyclines. (2) Methods: A MEDLINE search of the literature was performed, focusing on papers published in the last two decades. (3) Results and Conclusions: BRCA mutated tumors seem to show a higher response to anthracyclines compared to sporadic tumors and the severity of hand–foot syndrome and mucositis may be a predictive marker of PLD efficacy. CA125 can be a misleading marker of clinical response during treatment with anthracyclines, the response of which also appears to depend on OC histology. Immunochemistry, in particular HER-2 expression, could be of some help in predicting the response to such drugs, and high levels of mutated p53 appear after exposure to anthracyclines and impair their antitumor effect. Finally, organoids from OC are promising for drug testing and prediction of response to chemotherapy.

## 1. Introduction

With more than 313,000 new cases per year, ovarian cancer (OC) is the eight most common cancer in women worldwide. Despite substantial improvement in its diagnosis and treatment, it is still the most lethal among gynecological cancers, accounting for almost 207,000 deaths each year [1]. Cytoreductive surgery and platinum-based chemotherapy is the standard treatment. Three-weekly carboplatin/paclitaxel remains the standard of care as first-line chemotherapy, with the addition in selected patients of molecularly targeted therapies, such as poly (adenosine diphosphate-ribose) polymerase (PARP) inhibitors or the antiangiogenic drug bevacizumab. The majority of patients will, anyway, relapse, within the first two years and the prognosis and probability of response to second-line chemotherapy is greatly influenced by the platinum-free interval (PFI), despite efficacy of some targeted therapies being independent from PFI [2].

Among the numerous therapies available nowadays are anthracyclines, which represent one of the cornerstones of both first and subsequent-lines of chemotherapy in OC [3]. These drugs derive from the bacterium Streptomyces peucetius var caesiusare and are grouped under the class of antitumor antibiotics. They act mainly by inhibiting DNA topoisomerase-2 [4], causing cells to undergo apoptosis; moreover, they produce free radicals which damage cell membranes, proteins and lipids [5]. They also cause DNA alkylation and operate through DNA cross-linking, plus interacting with DNA strand separation and helicase activity. Finally, they also appear to have a direct effect on cell membranes [6].

Among anthracyclines, doxorubicin, Pegylated Liposomal Doxorubicin (PLD) and epirubicin are the most used in OC. In particular, doxorubicin is a prodrug and its encapsulated version, PLD, presents reduced toxicity and a better pharmacokinetic profile [7]. In fact, liposomes’ size of about 100 nm prevents their entrance into tissues with tight capillary junctions, such as the myocardium, and gives PLD slower plasma clearance and higher tissue concentration [8].

PLD is overall the most used anthracycline for its particularly advantageous pharmacokinetic profile. This drug was first approved in 1999 by the FDA and in 2000 by the EMA as a single agent for the treatment of advanced OC after the failure of first-line platinum-based chemotherapy. Moreover, the results from phase III trials suggest a further role for PLD in the salvage setting and in second- or front-line treatment in combination with other therapeutic drugs [9].

Congestive heart failure is the most relevant dose-dependent side effect of anthracyclines, with about 26% of patients experiencing cardiotoxicity [10]. The mechanism for cardiotoxicity is mainly due to the action of free radical damage through lipid peroxidation over the myocytes [11] and the toxicity rises as the plasmatic peak of anthracyclines increases [12]. With PLD, however, the overall risk is significantly lower compared to doxorubicin (HR: 3.16, *p* < 0.001) [13] and in carefully selected patients more than six cycles of PLD are well tolerated [14]. Up to now, re-challenge treatment with PLD in OC is not the standard of care: in breast cancer, anyway, retreatment with anthracyclines seems possible, with limited cumulative toxicity [15].

Another relevant side effect of anthracyclines is palmar–plantar erythrodysesthesia, which occurs in about 21.6% of the patients [16]. PLD has, in fact, a preferential concentration in the skin due to the polyethylene glycol coating and the incidence of this side effect significantly limits the PLD dose that can be administered if compared with conventional doxorubicin [17].

It is of extreme importance to select patients who would benefit the most from anthracyclines to maximize pharmacological efforts. The aim of the current narrative review is to provide a summary of the clinical and biological mechanisms involved in response to anthracyclines focusing on OC. We also discuss the potential implications of new therapeutic targets in anthracyclines activity.

## 2. Methods

A MEDLINE search of the specific, most relevant literature was performed, mainly focusing on papers published in the last two decades on the role of anthracyclines in ovarian cancer treatment. All findings within our search were combined into a narrative description, dividing it into different paragraphs based on the different topics we intended to explore. The main findings were discussed through the text. The recruited papers were published between 1982 and 2021. Keywords included in the search were: anthracyclines; Pegylated Liposomal Doxorubicin; BRCA mutation; chemotherapy; immunohistochemistry; immunotherapy; organoids; ovarian cancer; p53; response rate. Additional publications were identified via a systematic review of all reference lists within the publications retrieved from the MEDLINE search.

## 3. Results

### 3.1. Treatment Line and Platinum Free-Interval

In their systematic review, Lawrie et al. found no difference in PFS (HR: 1.01; 95% CI 0.85 to 1.19) or OS (HR: 0.94; 95% CI 0.78 to 1.13) between carboplatin/PLD (CD) and carboplatin/paclitaxel (CP) in first-line treatment of OC patients [18]. Given the reported difference in toxicity in the MITO-2 trial, Pignata et al. concluded that (CD) can be considered a reasonable alternative for first-line treatment, particularly in patients at high risk of neurotoxicity or wishing to avoid alopecia [19]. No survival benefit was, instead, found when adding PLD to (CP), and the triplet was associated with more hematological toxicity. So far, no randomized clinical trial has evaluated PLD as a single agent in the first-line setting, although PLD alone is advantageous in case of renal impairment, patient’s refusal of alopecia or platinum hypersensitivity [18].

In the recurrence setting, anthracyclines are available as a single agent in platinum-refractory or resistant patients, in combination with trabectedin in partially platinum-sensitive patients and in combination with platinum in platinum-sensitive tumors. PLD was introduced in the recurrence setting in 2001 after the publication of a randomized trial, showing its advantage over topotecan in platinum-resistant OC patients (PFS was 108 versus 71.1 weeks, respectively, *p* = 0.008) [9].

In the CALYPSO (CAeLYx in Platinum Sensitive Ovarian) trial the combination of CD in the recurrent setting was associated with better PFS compared to the standard CP arm (11.3 vs 9.4 months, respectively, HR: 0.821, *p* = 0.005) [20]. In partially platinum-sensitive patients, the hazard ratio for PFS was 0.73 (95% confidence interval: 0.58–0.90; *p* = 0.004 for superiority) in favor of CD, with a median PFS of 9.4 months (CD) and 8.8 months (CP) [21]. A more recent analysis in patients with a treatment-free interval > 24 months showed comparable efficacy of the two regimens in terms of PFS, however the authors concluded that the favorable risk–benefit profile recommends CD as a treatment of choice for these patients [22]. Furthermore, a considerable amount of patients will eventually experience hypersensitivity to platinum after multiple lines of chemotherapy: in the CALPYSO trial, reduced hypersensitivity was reported among OC patients treated with CD compared to the CP arm (18.8% versus 5.6%) [20].

In the OVA 301 trial, PLD plus trabectedin was associated with improved PFS over PLD alone in the second line treatment of fully platinum-sensitive OC patients (9.2 vs 7.5 months, respectively, HR: 0.73; 95% CI, 0.56–0.95, *p* = 0.0170) and partially sensitive disease (7.4 months versus 5.5 months, HR: 0.65; *p* = 0.0152) [23]. Patients treated with PLD/trabectedin combination showed longer time to subsequent therapy with platinum. This fascinating hypothesis that treatment with a non-platinum combination may prolong PFI and give more chance of success to further platinum therapy failed to be demonstrated in the INNOVATYION trial [24].

A recently published analysis of the phase III, randomized, open-label, multicenter trial comparing combination therapy of trabectedin/PLD versus PLD alone in the third-line treatment of recurrent OC showed that prior treatment with PLD in recurrent OC does not impact on the response rate, nor does it increase toxicities or negatively influence survival in both treatment groups [25].

In conclusion, intriguingly, the response to anthracyclines in OC, and in particular to PLD, is not influenced by the line of treatment or platinum-free interval.

### 3.2. BRCA-1-2 Status and Response to Anthracyclines

One mechanism of action of PLD is the induction of single-stranded and double-stranded DNA breaks through free radicals formation and direct intercalation into DNA, interfering with topoisomerase II-mediated DNA repair [6]. BRCA-1 and BRCA-2 are critical genes for homologous recombination, the favored way of repairing DNA double-strand breaks induced by chemotherapy; cells carrying BRCA mutation cannot use the homologous recombination system to fix DNA damage and undergo apoptosis more easily. It is well known that BRCA mutated tumors benefit the most from platinum-based treatment [26,27,28,29,30], which confers mutated patients a longer DFS and OS when compared to women with sporadic OC [31]. However, growing evidence suggests that mutated tumors are also more sensitive to other DNA damaging agents, such as PLD [32]. According to the literature, BRCA mutated OC is in general more responsive to the group of DNA damaging drugs and more resistant to antimicrotubule agents (e.g., taxanes), in comparison with sporadic tumors [33].

A phase II trial comparing the PARP inhibitor olaparib at two doses versus PLD in a population of BRCA-1/2-mutant patients with recurrent OC showed a greater than expected objective response rate to PLD [34]. In the retrospective cohort study by Safra et al., the response to doxorubicin correlated with OS (56.8 vs 22.6 months, 95% CI: 17.0–34.1, *p* = 0.002, in patients with and without BRCA mutation, respectively). No association with survival was, instead, found when considering the number of the line of treatment or platinum sensitivity [32]. Adams et al. reported that 56.5% of OC patients with BRCA mutation versus only 19.5% of non-mutated patients responded to PLD (*p* = 0.004), with increased PFS and OS for the first group, irrespective of platinum sensitivity [35].

If a different response to PLD treatment exists between BRCA-1 and BRCA-2 mutations is yet to be understood, however, the difference is expected to reproduce the one reported for platinum [36]. In the setting of OC, in fact, patients carrying a BRCA-2 mutation were found to have significantly higher responses to primary chemotherapy and improved platinum-free interval [37]. Historically, BRCA mutation was regarded as one single mutation, however, in recent years it has become clear that different sites of mutation in BRCA-1 gene confer different platinum and PARP inhibitors’ sensitivity profiles. In particular, mutations within exon 11 and those affecting BRCA-1 RING domain function may not confer any BRCAness phenotype at all [38]. Few studies have also indicated a role of mutation position in the BRCA-2 gene [39,40,41]. 

Secondary BRCA events seem to be correlated with the development of chemoresistance for both BRCA-1 and 2 mutated patients [42], and while patients carrying the BRCA-2 mutation continue to experience longer OS in comparison with the sporadic counterpart after five years from diagnosis, recent studies show that women with the BRCA-1 mutated gene fail to experience any survival advantage after ten years [43]. 

Hollis et al. investigated whether the BRCA-1/2 status influences the response rate to single-agent PLD in a histologically uniform cohort of high grade serous OC patients and found a superior response rate to PLD in OC patients harboring variants likely to affect the BRCA-1 or BRCA-2 function compared to the BRCA-1/2 wild-type population (36%, nine of twenty-five patients versus 12.1%, seven of fifty-eight patients; *p* = 0.016). An enhanced response rate was also seen in patients harboring the BRCA-1 SNP rs1799950 mutation, which is regarded to be detrimental for the BRCA-1 function (50%, three of six patients versus 12.1%, seven of fifty-eight patients; *p* = 0.044) [44]. 

Finally, Monk et al. recently reported that patients with germline BRCA-1/2 mutations appear to have a clinically relevant survival benefit with a combination of trabectedin/PLD versus PLD alone in platinum-sensitive recurrent OC. Patients with BRCA-1/2 mutations had a median OS of 34.2 months with trabectedin/PLD vs. 20.9 months with PLD (HR: 0.54, 95% CI:0.33–0.90; *p* = 0.016). In patients with BRCA-1/2 mutations and a 6–12 months PFI, median OS was 31.5 vs. 14.9 months, respectively, (HR:0.37, 95%CI:0.17–0.82; *p* = 0.011) [45].

PARP inhibitors as single agents have shown very modest activity in platinum-resistant OC patients in a BRCA-non selected population. The ongoing GEICO1601-ROLANDO trial is a protocol designed with the aim of assessing efficacy and safety of the combination of olaparib and PLD followed by olaparib maintenance in such setting [46].

### 3.3. Patients’ Clinical Characteristics and Sensitivity to Anthracyclines 

Median age at first diagnosis of OC is currently 63, with approximately one third of patients aged 70 or older. The incidence of each OC histotype varies with age. Elder women are largely under-treated and under-represented in clinical trials and they seem to have poorer outcomes compared to the younger counterpart [47]. In a subgroup analysis of the CALYPSO trial, similar to all patients in the study, carboplatin/PLD combination provided a better therapeutic response with similar PFS and less toxicity than carboplatin/paclitaxel in women older than 70 (median 74 years, range 70–82 years). When comparing elderly and younger patients, the first ones also experienced fewer grade ≥2 allergic reactions (*p* = 0.005) [48]. 

As a result of its expanded and extensive use, an increased incidence of carboplatin-associated hypersensitivity reactions (HSR) has been observed. Managing carboplatin allergy is an important challenge since up to 50% of patients who experience a mild to moderate HSR will discontinue therapy prematurely, even despite platinum re-challenge protocols. HSRs documented within case report forms and SAE reports were specifically analyzed in the Calypso trial. Patients randomized to the PLD-containing arm experienced significantly less HSRs than those who received CP (15.5% versus 33.1%, respectively, *p* < 0.001). Additionally, significantly fewer severe allergic reactions (>grade 2) were observed in patients in the CD arm than in the standard CP arm (2.4% versus 8.8%, respectively (*p* < 0.001). The greatest probability of reduced HSRs in the CD arm might be a protective immune effect induced by PLD. Pegylation appears, in fact, to lower the immunologic response by steric masking of antigenic sites, thereby preventing immune recognition of the therapeutic protein as foreign element [49].

Hand–foot syndrome (HFS) induced by chemotherapy with anthracyclines and molecule-targeting drugs seems to be correlated with treatment efficacy. In a retrospective analysis, when compared with patients with grade 0/1 HFS and oral mucositis, patients with grade 2–4 toxicity (*n* = 9, 33.3%) had a significantly higher clinical benefit (11.1% vs 77.7%; *p* < 0.001) and longer median OS (3.7 months vs. 20.8 months; *p* < 0.001). The authors concluded that the severity of HFS and mucositis may be a predictive marker of PLD efficacy. The prevention and management of HFS and mucositis are important in order to avoid treatment discontinuation.

### 3.4. CA125 Kinetics during Treatment with Anthracyclines

In a small cohort of platinum-resistant patients (*n* = 50), Oaknin et al assessed CA125 fluctuation patterns in responder and non-responder recurrent OC patients receiving PLD and concluded that, according to the predictive positive value of CA125 variation over time during treatment with PLD, 20% of the responders would be identified as non-responders (*p* = 0.025). Discontinuation of PLD therapy before cycle three may exclude some patients who will, instead, benefit from anthracyclines [50]. Similarly, Lee et al., comparing carboplatin-PLD (CPLD) with carboplatin—paclitaxel (CP) found fewer CPLD patients with an early decline of CA125 (161 [37.4%] vs. 233 [51.2%], *p* < 0.001) or an early response (146 [33.9%] vs. 176 [38.7%], *p* = 0.14) compared with CP patients. The PFS for CPLD patients did not statistically change after adjustment for early decline (adjusted HR = 0.80, 95% CI = 0.68 to 0.94, *p* = 0.007). These findings suggest this marker is not a good surrogate for treatment benefit with anthracyclines and its variations may not be faithful to the real efficacy of the ongoing treatment; CA125 variations should therefore not be misinterpreted, with premature treatment discontinuation in absence of other signs of disease progression [51]. On the other hand, Yuan et al reported lower CA125 levels at baseline and a significant reduction after the first cycle to be predictive factors for PLD efficacy; such markers also statistically correlated with ORR [52].

### 3.5. Anthracyclines and Ovarian Cancer Histology

OC is a heterogeneous disease that includes different histologies with distinct aetiologies and precursors (especially for low grade and high grade tumors). In OC histology with specific molecular patterns are relevant for choosing the best treatment, particularly in the maintenance setting [53]. It is, in fact, well known that different histologic subtypes present different chemoresistance profiles [54]. In their study, Holloway et al. showed a significant association between drug resistance and survival outcomes in OC patients, predicted by the in vitro Extreme Drug Resistance Assay [55].

In the literature, even when adjusting for other factors, the tumor histotype is widely associated with survival, partly due to different responsiveness to treatment. A SEER data analysis conducted in 2019 reported low-grade serous and endometrioid OC to have the most favorable outcomes, independently of stage, and reported definitely higher mortality for carcinosarcoma and distant-stage mucinous and clear cell OC [56,57]. In the past there has been some inconsistency among trials on histotype-specific survival, however, such studies presented many limitations. The majority of them were published prior to the 2014 WHO guidelines when the current knowledge of OC pathogenesis was unknown and histotype-specific survival was often not presented by stage.

In a large study conducted on this topic, Cloven et al. found significant differences in in vitro drug resistance between different histologic subtypes among 5195 OC patients [58]. As reported by the authors, overall, doxorubicin showed the highest incidence of drug resistance (40% of all tumor cells). However, when compared to papillary serous tumors, mucinous, endometrioid and clear cell tumors were significantly less resistant to doxorubicin. Possibly, therefore, such histologic subtypes may benefit most from the incorporation of anthracyclines in the treatment regimens and histology more than the tumor grade can help to predict the response to chemotherapy [59].

The tumor histotype and grade remain predictors of survival even after adjustment for stage and other factors, contributing to biological dissimilarity among different OC histotypes. Therefore, it remains imperative that we recognize OC as a set of distinct diseases and not a single entity to target the unique features of each histotype with the adequate treatment.

### 3.6. Gene Expression and Immunochemical Parameters

The prognostic implication of expression of Her-2 neu in OC is still debatable. Some studies proved impaired survival outcomes with Her-2 neu overexpression, while others found no survival correlation [60,61,62,63]. In their study, Cloven et al. demonstrated an overall expression of Her-2 of about 16%, coherent with previous studies. The authors reported that clear cell OC histotype is the one showing the highest levels of Her-2 neu expression and that this correlated with the lowest resistance to doxorubicin among all subtypes [58]. This finding is consistent with other studies in breast cancer where Her-2 neu expression levels were associated with a response to anthracyclines. Di Leo et al. reported in a study of 430 breast cancer that those with HER2-amplified tumors treated with anthracyclines had improved survival over those treated with cyclophosphamide, methotrexate, and 5-fluorouracil. No difference in terms of outcome with anthracyclines was seen in HER-2-non-amplified patients. The authors conclude that regimes containing anthracyclines could be more effective in patients with HER-2-amplified tumors [64].

As previously said, anthracycline antibiotics achieve their cytotoxic effects though a number of mechanisms. A principal mechanism is their ability to intercalate into DNA, bind DNA topoisomerase II and induce DNA cleavage in an ATP-dependent manner [5]. Type 2 topoisomerase alpha (TOP2A) gene is located on the locus q21 of chromosome 17, close to the HER-2 gene, and is responsible for coding TOP2A. Several retrospective analyses have suggested a correlation between TOP2A status and response to anthracyclines in breast cancer, both in neoadjuvant and adjuvant treatment. Conversely, in OC few studies have investigated the prognostic and predictive role of TOP2A with heterogeneous results [65]. Erriquez et al. demonstrated that TOP2A gene copy number is associated with protein overexpression and correlates with the activity of PLD in a small series of 38 platinum resistant OC patients and patients-derived xenografts (PDXs) [66]. Ghisoni et al. assessed the value of TOP2A expression by immunohistochemistry as a predictive marker of response to PLD-based therapy in patients with relapsed platinum resistant or partially platinum-sensitive OC. Patients with TOP2A expression > 18% treated with PLD as monotherapy achieved longer time to progression compared with PLD-doublet therapy (*p* = 0.05). The authors concluded that TOP2A status might predict activity of PLD in patients with relapsed platinum resistant or partially platinum-sensitive OC [65]. 

Finally, Perrone et al. performed a prospective-retrospective biomarker study within the MITO2 trial, a randomized multicenter phase three trial comparing carboplatin/paclitaxel and carboplatin/PLD as first line treatment [67]. Sixteen biomarkers (pathways of adhesion/invasion, apoptosis, transcription regulation, metabolism, and DNA repair) were studied in 229 patients, in a tissue microarray. Statistically significant interactions with treatment were found for DNA-dependent protein kinase (DNA-PK) and phosphorylated acetyl-coenzymeA carboxylase (pACC), both predicting worse outcome for patients receiving carboplatin/paclitaxel. These data show that in the presence of DNA-PK or pACC overexpression, carboplatin/paclitaxel might be less effective than carboplatin/PLD as first line treatment of OC patients. Further validation of these findings is warranted.

### 3.7. TP53 Mutation and Resistance to Anthracyclines

Information on the molecular background of OC has been assimilated in its disease management. Mutation of the nuclear transcriptional regulator p53 is present in about half of all types of human malignancies and is responsible for tumor progression mainly through a loss of function, although also in an oncogenic manner [68]. Cell-cycle arrest and subsequent apoptosis are the main consequences of wild-type p53 and the result of its mutation generally consists in higher rate of metastasis formation and increase in chemoresistance [69,70,71]. Prognostic role of TP53 mutation in OC is still debated [72,73,74]. Pathogenic TP53 mutations have been recognized in 95% of high grade OC patients [75,76], with more than different 2329 kinds of mutation identified [77]. Higher prevalence of TP53 mutations are found in patients with BRCA-1 mutation [78], as such genomic alteration induces a selective pressure against wild-type TP53 to continue with proliferation after DNA damage [79]. In their study, Bug et al. showed how the levels of mutant p53 are increased in cancer cells after exposure to doxorubicin and daunorubicin [68], mainly through activation of DNA damage-responsive kinase ataxia telangiectasia mutated [80]. This undesired effect of increase in mutant p53 levels linked to anthracyclines treatment seems to impair their antitumor effect [81]. Several authors have shown that some of p53 mutations, and in particular those affecting the domains L2/L3, are related with resistance to anthracyclines in breast cancer patients [82,83,84,85]. These results may explain the lack of efficacy of treatment with anthracyclines in patients harboring some p53 mutations. TP53-induced resistance seems also to correlate with anthracyclines in non-gynecologic cancers, as reported by Pandey et al. for bladder cancer [86]. Since TP53 gene mutations often go together with conformational changes in the conformation of p53 protein, some molecules seem promising to restore the original structure of the protein and rebuild the wild type function (PRIMA-1, MIRA-1 and some derivatives of the thiosemicarbazone family) [87]. In order to select patients who benefit the most from treatment with anthracyclines, improving our knowledge on the implication of p53 mutations should be pursued as a matter of the upmost importance. 

### 3.8. Immune System Expression and Anthracyclines

It is well recognized that OC displays immunologic features that provide a rationale for the use of immunotherapy. In the last few decades, there has been growing interest in the blockade of the PD-1/PD-L1 pathway and in chemotherapy drugs that enhance immunogenic cell death [88,89,90]. As tested in mice, anthracyclines are the only drugs that provide enhanced immunity by downregulating PDL-1 expression on the surface of the cell and upregulating its expression in the nucleus. Casares et al. showed that such drugs cause antitumor immune response by caspase activation and apoptosis that leads to eradication of the remaining tumor cells [91]. Fucikova et al., among others, reported that anthracyclines were able to induce immunogenic cellular death by inducing the expression of several immunogenic factors in ovarian cancer cell lines [92]. 

In preclinical models, the association of checkpoint inhibitors with PLD was correlated with increased efficacy compared to checkpoint inhibitors alone and several immunotherapy trials have selected anthracyclines for combination therapy among all possible chemotherapeutic drugs in light of their immunogenic potential. In particular, doxorubicin, in combination with an anti-PD-L1 antibody, proved to decrease regulatory T cells and increase the amount of CD8+, and improved survival [93]. The recent literature provides solid evidence for a significant relationship between CD8+ TILs and high-grade serous OC survival [94] and studies using checkpoint inhibitors and PLD are becoming of central value in OC: among others, in the JAVELIN Ovarian 200 trial, Avelumab was tested in OC patients randomized to receive avelumab as monotherapy, avelumab and PLD or PLD alone. 

Further research is needed to investigate if PDL-1 expression may correlate with anthracyclines response in OC. Anyway, a solid link between antitumor immune response and higher sensitivity to chemotherapy has been reported in breast cancer. In several studies, high levels of endogenous tumor immunity represented by tumor-infiltrating lymphocytes (TILs) have been correlated with a higher response rate to chemotherapy [95] and a more favorable prognosis [96,97,98,99]. More recently, in particular, TILs have been associated with a satisfactory response to anthracyclines in a large group of 1058 patients, concluding that they potentially predict sensitivity to anthracycline-based therapy [100]. In their study, West et al. demonstrated that the clinical benefit of neoadjuvant chemotherapy was restricted to breast cancer patients with a high number of TILs, whereas no association was found for such patients after chemotherapy with cyclophosphamide, methotrexate and fluorouracil [96].

Previous studies have shown that BRCA-1-deficient OC patients are more likely to display high levels of TILs and display an enrichment of immune response genes [101]. OC with high levels of T cell infiltration have a superior clinical outcome, thought to be secondary to an improved anti-tumor immune response [102]. A recent study suggested that PLD may enhance the immune response in BRCA-1-deficient tumors, and this may contribute to the improved benefit of PLD seen in BRCA-1/2-aberrant tumors [103].

### 3.9. MiRNAs

Recently, it has been shown that microRNAs (miRNAs) influence messenger-RNA (mRNA) post-transcriptional control and can contribute to human carcinogenesis. Boren et al. explored the role of miRNAs, and their predicted mRNA targets, in recurrent OV (OVCA) in vitro response to chemotherapy [104]. The expression of 335 unique miRNAs was measured in 16 OVCA cell lines and the sensitivity of these cell lines to six commonly used chemotherapeutic agents (cisplatin, doxorubicin, topotecan, paclitaxel, docetaxel, and gemcitabine) was evaluated. Twenty-seven miRNAs were found to be associated with the response to the one or more of the six salvage chemotherapies tested (*p* < 0.05) and seven miRNAs were associated with doxorubicin-response. Seven miRNAs were associated with the response to more than one chemotherapy drug, including miR-213 (doxorubicin, gemcitabine), miR-181a (doxorubicin, gemcitabine), miR-181b (doxorubicin, gemcitabine) and miR-520f (doxorubicin, cisplatin). Predicted targets of these miRNAs included 52 mRNAs, previously reported to be associated with chemo-responsiveness, and which are also involved in functional biologic pathways that influence cancer cell cytotoxicity, carcinogenesis, cell mitosis, p53 signaling, and tumor cell growth and invasion. Evaluating a previously reported 35-gene signature, 8/76 (11%) genes in a doxorubicin-response predictive signature were found to be predicted gene targets of the identified miRNAs. Dysregulation of microRNAs is then apparently involved in ovarian carcinogenesis and modifies the prognosis of OC patients. Such a field of research represents, to date, an intriguing therapeutic target that may help to predict the response to chemotherapy. However, there is still the need for further research to finally and concretely improve patients’ outcome.

### 3.10. Organoids

Despite initial promising results, most advanced OC patients will finally develop chemoresistance and about 90% of patients will finally die of disease [105]. The molecular nature underlying drug resistance remains, however, poorly understood [106] and there are no reliable markers of prediction of response. A particularly heterogeneous tumor burden at chemotherapy initiation might play a relevant role in the development of drug resistance in OC patients.

In recent years, an advance in molecular and clinical oncology in the light of personalized medicine has been a growing interest for organoids. They are in vitro, microscopic, three-dimensional structures grown from stem cells resected from tumor biopsy, dissociated through mechanical and enzymatic mechanisms and complemented with several growth factors and hormones. Such structures reproduce key features of patients’ tumor characteristics and give the unique possibility to test chemotherapy response and better tailor patients’ therapies [107], through interrogation of specific biomarkers [108]. Organoids appear superior to conventional cell lines for drug testing and have great potential for the screening of different anticancer drugs, with intriguing data also showing for OC patients. Several studies have proved the reliability of such organoids in reproducing the original tumor characteristics with great help in the understanding of the mechanisms of chemoresistance in recurrent disease [109,110].

No specific and mature data are available for anthracyclines yet. However, a recent study tested the effects of various standard chemotherapies on organoids, including doxorubicin. Curves reporting drug-response showed distinct sensitivities of the organoid lines for different drugs, indicating different efficacies of the distinct drugs on individual tumor organoid lines [107]. In conclusion, even though research still needs to be conducted, there are great expectations with regard organoids as they might be an ideal tool for precision medicine in OC (see Supplementary Material Table S1).

## 4. Discussion and Conclusions

Adriamycin, the second oldest anthracycline, was first investigated as a cure for OC in the 1970s, even before platinum, which nowadays is the milestone in the medical treatment of OC. Anthracyclines are antitumor antibiotics, one of the most potent and broadly effective classes of chemotherapeutic agents [5].

When choosing a second line treatment for an OC patient, clinicians can propose anthracyclines, and especially PLD, with a chance of response irrespective of PFI. Furthermore, anthracyclines may promote the activity of platinum by prolonging the PFI and preserve the sensibility to platinum by reducing the risk of hypersensitivity. These clinical benefits are especially important in patients with BRCA mutated tumors that benefit the most from platinum-based treatment. Mutated tumors are also more sensitive to other DNA damaging agents, such as PLD. One mechanism of action of PLD is the induction of double-stranded DNA breaks and BRCA 1/2 genes are critical for homologous recombination, the favored way of repairing DNA double-strand breaks. As a result, BRCA status is an actual marker to predict response to anthracyclines [32,33,34,35,37,45].

The induction of single and double-stranded DNA breaks by anthracyclines interferes with topoisomerase II-mediated DNA repair. Type 2 topoisomerase alpha (TOP2A) gene is responsible for coding TOP2A protein expression. In a retrospective analyses in three referral cancer centers, we demonstrated that the TOP2A gene copy number is associated with protein overexpression and correlates with the activity of PLD in a small series of OC and patients-derived xenografts [64]. Furthermore, we reported that TOP2A expression above 18% is associated with a higher probability of response to PLD in patients with platinum resistant or partially platinum-sensitive recurrent OC [65]. Although these results should be regarded as an hypothesis-generating attempt to identify a biomarker of response, we believe that only a multifactorial panel could be predictive of the response to PLD in ovarian cancer.

The type 2 topoisomerase alpha (TOP2A) gene is located on the locus q21 of chromosome 17, close to the HER2 gene. Clear cell carcinomas were found to have the highest Her-2 neu expression levels and the lowest incidence of extreme drug resistance to doxorubicin, consistent with studies that have linked Her2 expression with anthracycline response. Resistance to doxorubicin was also less frequent in mucinous and endometrioid carcinomas, suggesting that these histologic types may benefit most from incorporation of anthracyclines into their treatment regimens. Conversely, the high grade serous OC data supports the notion that patients with BRCA1/2 mutations display an increased response rate to PLD. Therefore, the response to anthracyclines may depend on ovarian cancer histology, however, it is influenced by linked factors, such as Her-2 neu expression or BRCA1/2 mutations [53,58].

Immune checkpoint inhibitors are under investigation in the treatment of cancer. OC is characterized by PD-L1 expression on tumor cells and tumor-infiltrating lymphocytes, however OC cells have a lower frequency of PD-L1 expression compared with established immunosensitive tumors. Anthracyclines have a range of potentially immunogenic effects against tumor cells suggesting a synergistic activity with immunotherapy. As a consequence, clinical studies combining a checkpoint inhibitor with PLD in OC are of major interest [91,103].

Patients can benefit from anthracyclines treatment at any age, in different lines, irrespective of PFI, with the reduced risk of hypersensitivity to platinum and improvement of their quality of life. We learned so much about PLD in OC by the Calypso trial and subsequent analyses. The sub-study on elderly patients, which had no equivalent in the literature when it was published, indicated age as a non-limiting factor of the efficacy of PLD [48]. The carboplatin/PLD regimen was safe and showed a better tolerability profile than carboplatin/paclitaxel, especially for sensory neuropathy and alopecia, two issues of outstanding importance in the care of elderly patients. At every age, quality of life should be weighed in the balance in the choice of the medical treatment of OC, both in first and subsequent-lines of chemotherapy.

In the field of research, miRNAs and their target mRNAs seem to be associated with a response to anthracyclines in OC and represent, to date, an intriguing therapeutic target [104]. In recent years, growing interest has been raised for organoids, which reproduce the original tumor characteristics and appear very promising for a better understanding of chemoresistance and predict the response to anthracyclines [111].

## Supplementary Materials

The following supporting information can be downloaded at: https://www.mdpi.com/article/10.3390/ijerph19074260/s1, Table S1: Summary of the main findings.
