# Supplementary material for: Predicting Response to Anthracyclines in Ovarian Cancer
Source: Int J Environ Res Public Health. 2022 Apr 2;19(7):4260. doi: 10.3390/ijerph19074260 (PMC8998349; doi:10.3390/ijerph19074260)
Supplement: Supplementary file 1 [file ijerph-19-04260-s001.zip › ijerph-1632866-supplementary.pdf]

Table S1. Summary of the main findings.

**Treatment line and platinum free-interval**	FIRST LINE SETTING<br>1. No difference in PFS or OS between carboplatin/PLD and carboplatin/paclitaxel in first-line treatment<br>2. No survival benefit in adding PLD to carboplatin/paclitaxel, but more hematological toxicity<br>RECURRENCE SETTING<br>1. Comparable or better efficacy of anthracyclines over carboplatin/paclitaxel in recurrent disease<br>2. PLD plus trabectedin is associated with improved PFS over PLD alone	*Lawrie TA et al, 2013*<br>*Pignata S et al, 2011*<br>*Pujade-Lauraine E et al, 2010*<br>*Colombo N et al, 2020*<br>*Monk BJ et al, 2010*
**BRCA 1-2 status and response to anthracyclines**	BRCA mutated OC is more responsive to the group of DNA damaging drugs (i.e. PLD) and more resistant to antimicrotubule agents in comparison with sporadic tumors	*Safra T et al, 2011*<br>*Venkitaraman AR, 2009*<br>*Kaye SB et al, 2012*<br>*Adams SF et al, 2011*<br>*Hollis RL et al, 2018*<br>*Monk BJ et al, 2020*
**Patients' clinical characteristics and sensitivity to anthracyclines**	1. Carboplatin/PLD combination provides better therapeutic response with similar PFS and less toxicity and grade ≥2 allergic reactions than carboplatin/paclitaxel in women > 70y<br>2. PLD gives less hypersensitivity than carboplatin and paclitaxel<br>3. The severity of hand-foot syndrome and mucositis may be a predictive marker of PLD efficacy	*Kurtz JE et al, 2011*<br>*Joly F et al, 2011*
**CA125 kinetics during treatment with anthracyclines**	CA125 marker is not a good surrogate for treatment benefit with anthracyclines and its variations may not be faithful to the real efficacy of the ongoing treatment (usual later decline than with carboplatin-paclitaxel)	*Oaknin A et al, 2010*<br>*Lee CK et al, 2011*
**Anthracyclines and ovarian cancer histology**	When compared to papillary serous, mucinous, endometrioid and clear cell tumors are significantly less resistant to doxorubicin and benefit most from incorporation of anthracyclines in treatment regimes	*Cloven N, 2004*<br>*Shimizu Y et el, 1998*
**Gene expression and immunochemical parameters**	1. Her-2 neu expression levels are associated with response to anthracyclines<br>2. TOP2A expression by immunohistochemistry is a predictive marker of response to PLD-based therapy in relapsed platinum resistant or partially platinum-sensitive OC	*Cloven N et al, 2003*<br>*Ghisoni E et al, 2019*
**TP53 mutation and resistance to anthracyclines**	Increase in mutant p53 levels linked to anthracyclines treatment seems to impair their antitumor effect	*Bush JA et al, 2002*<br>*Chrisanthar R et al, 2008*
**Immune system expression and anthracyclines**	1. Relevant immunogenic potential of anthracyclines that may enhance immunogenic cell death by downregulating PDL-1<br>2. PLD seems to enhance the immune response in BRCA 1-deficient tumors	*Casares N et al, 2005*<br>*Mantia-Smaldone G et al, 2014*
**MiRNAs**	Different miRNAs were found to be associated with doxorubicin-response and can help to predict response	*Boren T et al, 2009*
**Organoids**	Microscopic, three-dimensional structures grown from stem cells can reproduce key features of	*Maenhoudt N et al, 2020*

		patients' tumor characteristics and test chemotherapy response for each patient	
